# Colonic Resection in an 8-Year-Old Girl with Intractable Functional Constipation and Diffuse Colonic Dysmotility and Failed Antegrade Flushes

**DOI:** 10.1055/a-2212-0411

**Published:** 2024-01-31

**Authors:** Gabriella Danziger, Thomas O. Xu, Teresa Lynn Russell, Laura Tiusaba, Jennie Yun, Marc A. Levitt, Andrea Badillo

**Affiliations:** 1 Division of Colorectal and Pelvic Reconstruction, Children's National Hospital, Washington, District of Columbia, United States

**Keywords:** functional constipation, idiopathic constipation, antegrade continence enemas, colonic resection, diffuse colonic dysmotility

## Abstract

Optimal surgical management of patients with intractable constipation and diffuse colonic motility is not well defined. We present a patient with such a history, who ultimately achieved successful surgical management of constipation through a stepwise approach. An 8-year-old female presents with longstanding constipation and diffuse colonic dysmotility demonstrated with colonic manometry. She initially underwent sigmoid resection and cecostomy which failed and required diverting ileostomy. We initially proceeded with an extended resection, colonic derotation (Deloyers procedure), and neo-appendicostomy (neo-Malone) which resulted in successful spontaneous stooling for 1 year. Her constipation recurred and she subsequently underwent completion colectomy with ileorectal anastomosis given that she previously demonstrated ability to stool independently. Six months from surgery the patient continues to stool daily with assistance of fiber and loperamide. This case highlights a stepwise surgical approach to managing constipation due to diffuse colonic dysmotility and demonstrates that diffuse dysmotility may benefit from an upfront subtotal resection; however, it is crucial to assess a patient's ability to empty their rectum prior.

## Introduction

Intractable functional constipation (FC) is often difficult to manage in children who have failed medical treatments. Surgical options vary in their efficacy in providing patients with a successful treatment option. 1 2 3 4 Antegrade continence enemas (ACEs) present a suitable option for many patients to implement a stooling routine and bridge the child to daily continence. 1 5 6 However, the optimal treatment for a patient with diffuse colonic dysmotility remains unclear. In some cases, an ACE is used for such patients, but these often fail. 6 7 At this point, a colonic resection may be the best option to avoid a permanent ostomy. 7 8 The case we present assesses treatment of refractory FC in such a patient with diffuse colonic dysmotility.

## Case

An 8-year-old female initially presented with severe constipation beginning at the age of 1 year to another provider. She has a history of anxiety and has visited the gastroenterologist since the age of 2. At 6 years of age, the patient underwent an anorectal manometry, which showed an intact rectoanal inhibitory reflex, an exam under anesthesia, and full-thickness biopsy, which showed ganglion cells and ruled out Hirschsprung disease. The patient subsequently underwent empiric botulinum toxin injection into the anal sphincters, a cecostomy, and sigmoid resection. Three months later, an ileostomy with mucous fistula was created due to ongoing symptoms despite antegrade flushes.

**Fig. 1:**
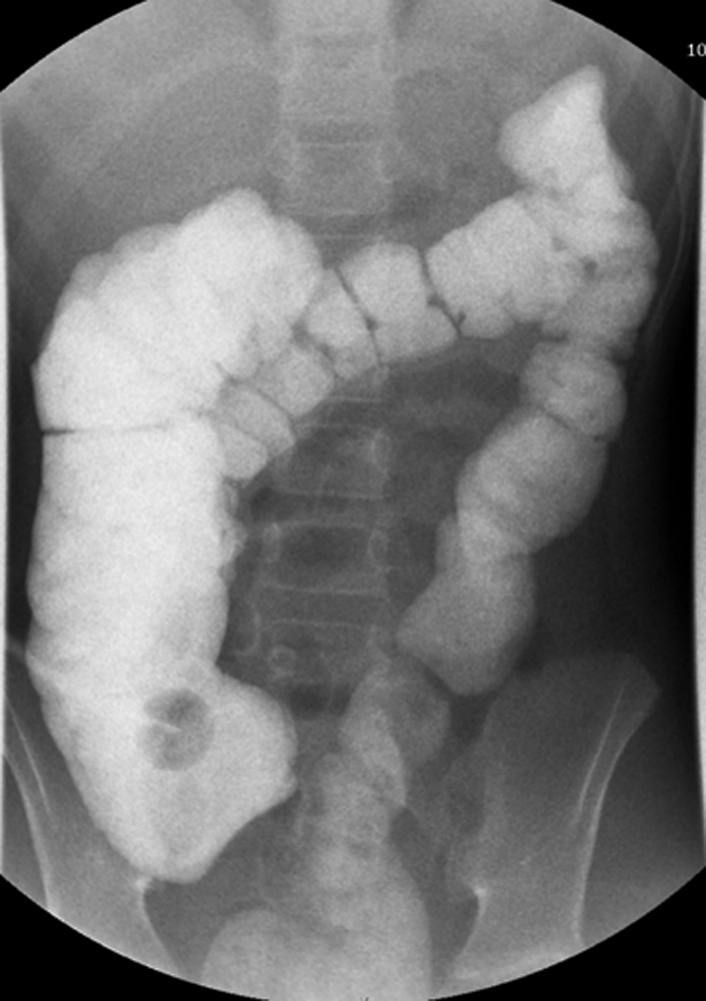
Contrast enema showing a distended right colon.

One year after her ileostomy, the patient was referred to our center for treatment. She was suffering from a prolapsed stoma and wanted the stoma closed. She required a nasogastric tube for hydration purposes. An ileostomy revision and cecostomy closure were performed. Colonic and (repeat) anorectal manometry testing showed that the patient had colonic dysmotility throughout the colon with no high-amplitude propagating contractions (HAPCs) present, and no signs of pelvic floor dyssynergia. She had high external sphincter resting pressures. A contrast enema was done which showed a dilated right colon and a normal transverse, left, and sigmoid colon with haustral markings seen indicating some peristalsis ( Fig. 1 ).

To remove the ileostomy, 6 months later, we performed an extended colon resection of the left and transverse colon, a right colon derotation (Deloyers procedure), and a neo-appendicostomy (neo-Malone) was placed in the right lower quadrant. The patient was noted to have a prior appendectomy (due to presence of staple line) intraoperatively which necessitated the creation neo-Malone. This was performed at another institution and we did not have any documented history of appendectomy at the time. For the colorectal anastomosis, a hand-sewn anastomosis was performed on the rectum at the level just proximal to the peritoneal reflection. Postoperatively, she experienced fever and leakage at the neo-Malone site and this was surgically closed. Following discharge, she experienced a normal stooling pattern without reliance on medications or enemas for 1 year.

**Fig. 2:**
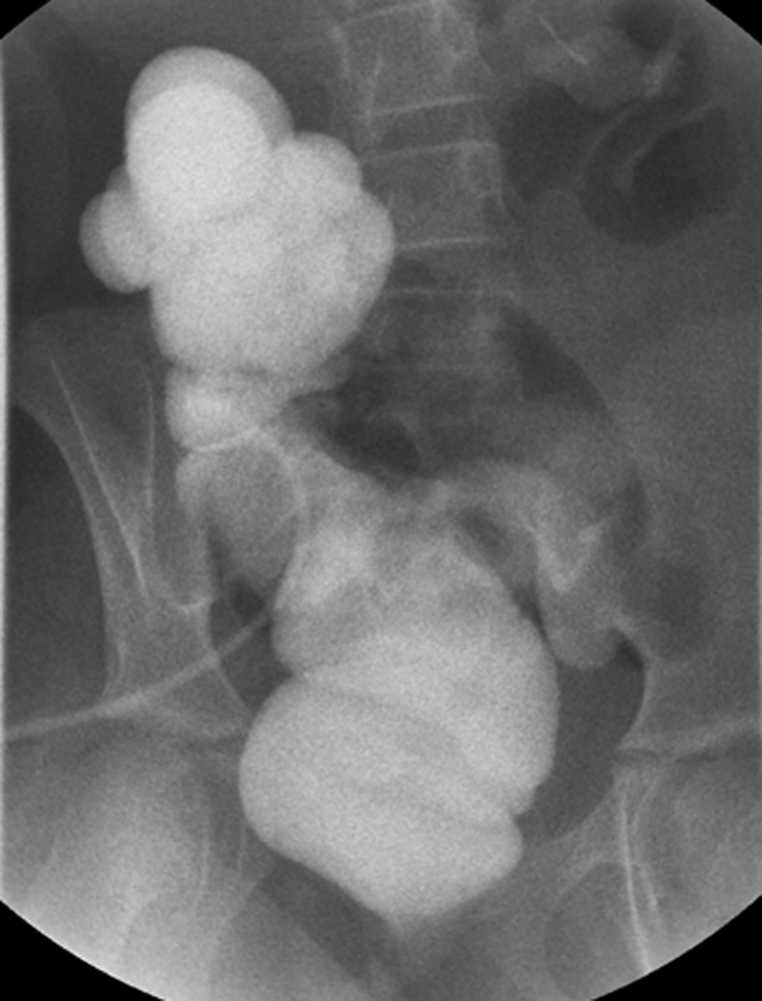
Contrast enema showing a dilated right colon without an anastomotic stricture.

One year later, the patient returned to our clinic with recurrent symptoms of constipation. She began taking 30 mg of senna daily. Rescuing of the neo-Malone site was attempted but could not be found so a cecostomy tube was placed instead. The patient commenced antegrade flushes. Accumulation of stool in the right colon was also noted in an updated contrast enema ( Fig. 2 ).

Because of failure of laxatives and of antegrade flushes, we decided to do a completion colectomy and performed an ileorectal anastomosis. The ileorectal anastomosis was made using an end-to-end stapler just distal to the prior coloanal anastomosis, at the proximal rectum at the level of the peritoneal reflection. Final pathologic review of the right colon specimen and anastomosis confirmed presence of ganglion cells throughout. We felt confident that she could empty her rectum on her own given that she was able to do this for 1 year. After this surgery, she is doing well with a constipating diet, empties daily, and has bowel control for at least 6 months. She unfortunately developed *Clostridium difficile* colitis which has since complicated her bowel management.

## Discussion

After multiple interventions it appears that the patient presented has arrived at a surgical solution for her intractable constipation with diffuse colonic dysmotility. We felt this case was instructive given this arduous stepwise road to her current anatomy and believe an initial resection should have been considered given the motility state of her colon.

Deciding the best surgical options for the management of pediatric patients with diffuse colonic dysmotility who have failed less invasive options is difficult. 1 2 3 5 7 A total colonic resection is considered a last resort for such children, and whether the rectum should be preserved is a key question. 2 3 7 8 Preservation of the rectum can be beneficial as it preserves a reservoir for stool. Furthermore, subtotal colectomy avoids the unnecessary morbidity of a completion proctectomy, especially in those who have demonstrated a functioning rectum/pelvic floor. An ileoanal anastomosis on the other hand is a more complicated procedure and has been well established to have worse continence outcomes. Benefits of proctectomy and ileoanal anastomosis are more pertinent to those with inflammatory bowel disease due to risk of malignancy or leaving residual disease; however, this benefit is not as pertinent in this population of patients. 9 Gladman et al reports subtotal colectomy with retention of the rectum as the most successful procedure for adults with idiopathic constipation. 3 Success rates for ileorectal anastomosis in children with constipation vary with the highest success rates being reported up to 70%. 1

Success of an ileorectal anastomosis (as opposed to ileoanal) is, however, contingent on a functioning pelvic floor as was demonstrated by this case. A key question in such patients is whether the rectum will empty spontaneously which gives insight into their pelvic floor function. Given this patient's history of normal stooling following her initial colon resection, we concluded that she has good pelvic floor function and that there would be no issues with emptying her rectal reservoir postoperatively. It is our belief that it is important to preserve the rectum and its role as a reservoir to maintain continence. 6 8 For patients that cannot clearly demonstrate the ability to empty their rectum, anorectal manometry with balloon expulsion test can be used as a more objective measure one's capacity to empty. For those with a poor balloon expulsion, pelvic floor physiotherapy can be used to improve their pelvic floor function. 6 10 11 12 The use of botulinum toxin, while effective for sphincter tightness, is unlikely to help in these patients although if there is concomitant sphincter pathology it should certainly be employed. Although a balloon expulsion test was not performed in this patient, in a similar future patient, we would advocate for keeping the ileostomy until it can be shown that the patient can successfully empty their rectum, that is, pass the balloon expulsion test.

Colonic manometry is a useful tool for assessing dysmotility and the need for a colon resection. 13 14 A study of 555 patients showed that abnormal colonic manometry is predictive of surgery, although the type of surgery was not included in the study so it cannot be directly predictive of the need for a colonic resection. 13 For pediatric patients with diffuse colonic dysmotility who have failed medical management of their constipation, controversy still exists on whether a colonic resection should be performed as the primary surgical treatment, or whether an antegrade option or an ostomy should be attempted first. 1 2 4 5 ACEs can help to improve colonic motility by normalizing HAPCs but are not well studied in this unique group of patients. 1 5 13 14

## Conclusion

This case demonstrates the clinical steps that were used for treating a patient with diffuse colonic dysmotility who failed every treatment until the entire colon was removed. If antegrade flushes do not work in a patient with diffuse colonic dysmotility (and they rarely do), a subtotal colectomy with ileorectal anastomosis is likely needed, but prior to this the patient must demonstrate the capacity to empty their rectum.
